# Bartonella Endocarditis Presenting as Recurrent Cerebral Mycotic Aneurysm

**DOI:** 10.7759/cureus.19969

**Published:** 2021-11-28

**Authors:** Osakpolor Ogbebor, Veena Pawate, Jean Woo, Kevin Kelly, Russell Cerejo, Nitin Bhanot

**Affiliations:** 1 Internal Medicine/Infectious Disease and Critical Care, Allegheny Health Network, Pittsburgh, USA; 2 Neurology, Allegheny Health Network, Pittsburgh, USA; 3 Infectious Disease, Allegheny Health Network, Pittsburgh, USA; 4 Vascular Neurology, Allegheny Health Network, Pittsburgh, USA

**Keywords:** warthin starry stain, cerebral angiogram, glomerulonephritis, focal fibrocellular crescent, intracranial hemorrhage, endocarditis, blood culture negative endocarditis, subarachnoid hemorrhage, bartonella henselae, cerebral mycotic aneurysm

## Abstract

Bartonella henselae is a known cause of culture-negative endocarditis, which can be difficult to diagnose without a high clinical suspicion as specific diagnostic testing is required.

We report the case of a 48-year-old male who presented with altered sensorium. A CT of the head showed left-hemispheric intracranial hemorrhage (ICH) likely secondary to ruptured left posterior cerebral artery (PCA) fusiform aneurysm seen on catheter cerebral angiogram, which was treated with endovascular embolization. The patient had a significant history of mitral valve prolapse; however, a transthoracic echocardiogram (TTE) was negative for any vegetation. Blood cultures were also negative. A year later, he presented with another ICH in the PCA territory and was found to have a new left distal PCA aneurysm, which was again treated with endovascular embolization. During that hospitalization, an echocardiogram showed myxomatous changes in the mitral valve with severe mitral regurgitation; however, blood cultures were negative. Further queries about the patient’s social history revealed that his spouse had been a cat owner in 2018, which prompted Bartonella henselae testing. The blood work showed elevated immunoglobulin G (IgG) titers for which he was placed on antibiotics. A follow-up catheter angiogram detected a new distal middle cerebral artery (MCA) M4 branch aneurysm treated with surgical clipping. The aneurysm tested positive for Bartonella henselae on polymerase chain reaction (PCR) testing. The patient subsequently underwent successful mitral valve replacement, which also was positive for Bartonella henselae on PCR testing; however, the Warthin-Starry stain was negative.

This case demonstrates how a comprehensive history along with persistent evaluation for the underlying etiology of cerebral aneurysms can lead to the diagnosis of Bartonella henselae endocarditis. Cerebral mycotic aneurysms are known complications of endocarditis; however, the underlying infection can be difficult to diagnose. Recognition of this culture-negative endocarditis is critical for the appropriate treatment and management of patients to prevent morbidity and mortality.

## Introduction

About 8.1% of all endocarditis cases are culture-negative, and Bartonella henselae is the most common cause behind most of them [1]. A high clinical suspicion is warranted to diagnose Bartonella endocarditis since its presentation may be subacute and may mimic non-infective disease processes. Additionally, it is important to pay attention to systemic complications as the presentation of endocarditis can be non-cardiac-related, like cerebral mycotic aneurysms. Comprehensive history and specific testing modalities, such as polymerase chain reaction (PCR), are of paramount importance to diagnose culture-negative endocarditis. In this report, we discuss the case of a patient with culture-negative endocarditis who presented with recurrent mycotic aneurysms.

## Case presentation

**Figure 1:**
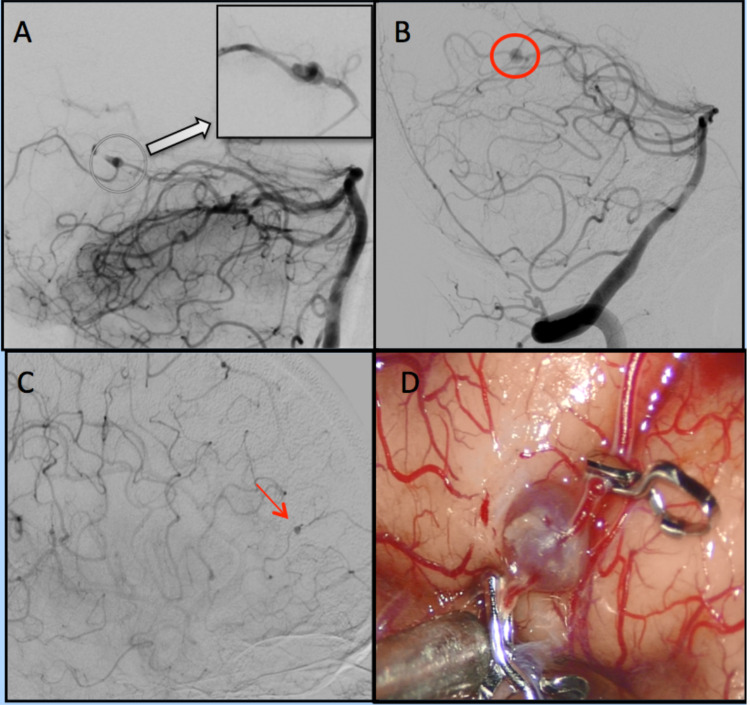
Images of cerebral mycotic aneurysm of the patient A. Angiogram showing peripheral left PCA fusiform mycotic aneurysm. B. Angiogram showing peripheral left PCA mycotic aneurysm. C. Angiogram showing peripheral right MCA mycotic aneurysm. D. Distal MCA aneurysm surgical trapping PCA: posterior cerebral artery; MCA: middle cerebral artery

A 48-year-old male presented to our hospital in 2018 with complaints of altered sensorium and syncope. On admission, a CT of the head showed a large intracranial hemorrhage (ICH) centered in the left occipito-parietal area with extension into the intraventricular space. The patient subsequently underwent craniotomy and hematoma evacuation. Catheter cerebral angiogram revealed a fusiform aneurysm measuring 5 mm by 2 mm, arising from the distal P3 segment of the left posterior cerebral artery (PCA) (Figure 1A), which was treated with endovascular embolization. A transthoracic echocardiogram (TTE) revealed moderate mitral regurgitation with the anterior mitral leaflet appearing thickened and redundant, but without evidence of any vegetation. Multiple sets of blood cultures returned negative.

Two years after his initial hospitalization, he was readmitted due to headaches. A head CT scan showed intraventricular hemorrhage predominantly in the lateral ventricle. On catheter cerebral angiography, he was found to have a new left distal P3 aneurysm measuring approximately 4.2 mm by 4 mm by 3.6 mm (Figure 1B). The aneurysm was treated by repeat endovascular embolization. During that hospitalization, a transesophageal echocardiogram showed myxomatous changes and thickening of the anterior mitral leaflet, measuring 1.6 cm by 1.5 cm, and severe mitral regurgitation. Blood cultures were repeated, but once again remained sterile. This patient’s workup additionally revealed an acute kidney injury and hypocomplementemia. Renal biopsy confirmed an immune complex-mediated focal segmental necrotizing glomerulonephritis with a focal fibrocellular crescent.

These findings raised the concern for an underlying endovascular infection and prompted workup for culture-negative endocarditis. Further social history review revealed that the patient’s spouse had owned a cat at the time of his original illness in 2018. Serologies submitted for Coxiella, Brucella, and Bartonella quintana were negative; however, immunoglobulin G (IgG) titers for Bartonella henselae were reactive at 1:2560. The patient was started on a regimen of doxycycline and rifampin while awaiting brain and cardiothoracic surgical intervention. Aminoglycoside therapy was avoided in light of the patient’s acute kidney injury.

Two months after the second hospitalization, the patient was readmitted with congestive heart failure exacerbation. He underwent a catheter cerebral angiogram, which detected a new right middle cerebral artery (MCA) M4 branch aneurysm, measuring 1.8 mm by 1.7 mm (Figure 1C). He underwent surgery to trap the aneurysm (Figure 1D). Operative specimens were sent for broad-range bacterial PCR and sequencing, which was positive for Bartonella henselae. At that time, given the resolution of his previous acute kidney injury, gentamicin was added to doxycycline.

Eight weeks later, the patient underwent successful mitral valve replacement surgery. The operative specimen was negative on Warthin-Starry stain (silver-impregnated stain used to detect Bartonella) and bacterial culture. Pathology showed benign valve tissue, consistent with calcification and degeneration. Valve tissue PCR, however, was positive for Bartonella henselae. The patient continued to improve clinically and was prescribed an additional three-month course of doxycycline postoperatively.

## Discussion

A high index of clinical suspicion is warranted to diagnose culture-negative endocarditis. Detailed history and targeted testing are needed to identify pathogens of potential concern, such as Bartonella, that do not grow by routine blood culture techniques. In a study carried out at a referral center in France, Bartonella species were found to be responsible for 28% of culture-negative endocarditis cases [1]. Bartonella quintana and Bartonella henselae are responsible for most of the valvular infections [2]. Risk factors for Bartonella henselae endocarditis include contact with cats, cat fleas, and preexisting valvulopathy [2].

Besides causing endocarditis, Bartonella infections may manifest with several noteworthy extracardiac infections. Due to its ability to mimic other forms of systemic diseases, including pauci-immune vasculitis and glomerulonephritis [3], bartonellosis can elude all but the astute physician. Mycotic cerebral aneurysms are among the most concerning presentations, given their high rate of rupture and resultant mortality [4,5]. Our review of the literature has identified a few reported cases of Bartonella endocarditis associated with intracranial aneurysm (Table 1) [4,6,7,8]. Four out of five patients had Bartonella henselae infection while the other case was caused by Bartonella vinsonii. Three presented with neurological symptoms, but it was unclear why neuroimaging was performed in the other two cases. Mycotic aneurysms were frequently seen in the MCA territory. Classically, intracranial mycotic aneurysms are located in peripheral arterial branches [4]and are fusiform in morphology, as compared to berry aneurysms, which are more proximal, at branching points, and saccular in morphology. Similar to our case, recurrent intracranial aneurysms are not uncommon, and hence repeating catheter angiography is important in the workup and management of these patients [4]. An echocardiogram revealed significant valvulopathy in most cases, which may reflect the chronicity of the disease before a diagnosis is made.

Symptomatically, patients presented with an array of nonspecific symptoms and had elements of renal and cardiac failure. Garg and Khosroshahi have described a case of glomerulonephritis initially treated with steroids and cyclophosphamide [4]. Similar to our case, renal biopsy demonstrated necrotizing glomerulonephritis with crescents and pauci-immune staining, further highlighting the association of Bartonella infective endocarditis with pauci-immune vasculitis. Interestingly, over half of the cases of Bartonella-infective endocarditis have positive antineutrophil cytoplasmic antibody (ANCA), which further confounds this diagnosis [8]. Extracardiac manifestations such as glomerulonephritis and intracranial aneurysm may be the initial clinical manifestations; hence, there is a need for a high index of suspicion for underlying endocarditis in such instances.

Our case highlights the fact that newer testing modalities such as gene sequencing and PCR may be needed to make a microbiological diagnosis in patients with presumed culture-negative endocarditis. In diagnosing Bartonella endocarditis, valvular tissue is the ideal testing specimen, as the sensitivity of tissue PCR is 92%, compared to 58% for blood [9]. Combined serology with IgM and IgG has a sensitivity as high as 85% but may cross-react with Chlamydia species and Coxiella burnetii, thereby confounding the diagnosis [10]. Bartonella does not adequately take up Gram stain, but Warthin-Starry staining may also be negative, as was the case in our patient. Molecular testing such as PCR should be routinely utilized to make a reliable diagnosis in proper clinico-epidemiological settings.

**Table 1:** Summary of previously published cases of Bartonella endocarditis associated with intracranial aneurysm B. henselae: Bartonella henselae; GN: glomerulonephritis; MCA: middle cerebral artery; SAH: subarachnoid hemorrhage; CTA: computed tomography angiogram; MR: mitral regurgitation; MVR: mitral valve repair; AVR: aortic valve repair; PCR: polymerase chain reaction; AV: aortic valve; VW: von Willebrand; B vinsonii: Bartonella vinsonii; MRI: magnetic resonance imaging; AI: aortic insufficiency; PMHx: past medical history; SOB: shortness of breath

Author name, year	Age and gender	Symptoms	PMHx	Exposure to cat	Associated disease on presentation	Echo findings	CTA/MRI findings	Diagnostic test	Antibiotics	Valvular surgery	Neurosurgery
Garg and Khosroshahi, 2017 [4]	55 years, male	Fever, bilateral ankle swelling and petechial pruritic rash, shortness of breath and chest tightness, delirium	Alcohol-dependent, chronic smoker	Yes	Pauci-immune GN	Small mobile masses on the AVs with severe AI	4.1 x 4.4 x 5.1 x 1.3-mm fusiform aneurysm of the distal M3 branch of the MCA and a 7.2 x 6.9 x 13.3 x 4.6-mm fusiform aneurysm of the M2 branch of the MCA	PCR blood: B. henselae, B. henselae serology, IgG >1:800	Doxycycline 6 weeks, gentamicin 2 weeks	None	None
Lockrow et al., 2016 [6]	39 years, male	Seizure	Hypertension	No	Intraparenchymal hemorrhage, subdural hematoma	2 small mobile mitral valve vegetations	Distal right posterior cerebral artery 4.8 x 5.6-mm aneurysm	B. henselae serology, IgG 1:2014	Doxycycline 6 weeks, gentamicin 2 weeks	None	None
Varga et al., 2020 [7]	60 years, male	Headache	None	Yes		Moderate to severe MR and 8 mm x 9-mm vegetation on the anterior mitral valve leaflet	Left basilar SAH, CTA showed a 3-4-mm distal left MCA branch aneurysm	B. henselae serology, IgG 1:2014, PCR valve: B. henselae	Doxycycline 6 weeks, gentamicin 2 weeks	MVR	Endovascular embolization
Beckerman et al., 2020 [8]	21 years, male	Fever, night sweats, pulmonary edema	Bicuspid AV	Yes	Heart failure, renal failure, anemia	Thickened and dysplastic AV. Moderate to severe AI. Moderately reduced left ventricular systolic function No vegetations	MRI mycotic aneurysm, 2.5 mm x 2 mm in the right MCA	PCR blood: B. henselae	Doxycycline 6 weeks, and for life	AVR	MRI-guided stereotactic right frontotemporal craniotomy and clip ligation of a mycotic aneurysm
Beckerman et al., 2020 [8]	20 years, male	Chest pain, SOB	Noonan syndrome, asthma, VW disease	No	Heart failure, renal failure, anemia	Thickened mitral with severe stenosis. No vegetations	1.3 cm x 1-cm partially thrombosed left frontal MCA aneurysm	PCR blood: B. vinsonii	Doxycycline	MVR	CT-guided stereotactic left frontoparietal craniotomy with resection and ligation of the aneurysm

## Conclusions

The case highlights the subacute manifestation of Bartonella endocarditis, which is a relatively rare presentation in the form of cerebral mycotic aneurysms, and, most importantly, underscores the need for relevant history to guide targeted testing in elusive cases of culture-negative endocarditis. As this case highlights, it is not sufficient to just diagnostically test with Warthin-Starry culture but clinicians should take a step further with PCR testing to make an accurate diagnosis. Bartonella endocarditis is a treatable disease, making it even more crucial to reduce morbidity and mortality.
